# Influence of daytime of allogeneic stem cell transplantation on incidence of acute graft vs. host disease: a retrospective analytical cohort study

**DOI:** 10.1038/s41409-025-02689-w

**Published:** 2025-08-02

**Authors:** Hauke Wilcken, Klaus Kaier, Thomas Meyer, Miguel Waterhouse, Linda Gräßel, Jesus Duque-Afonso, Reinhard Marks, Ralph Wäsch, Jürgen Finke, Robert Zeiser, Kristina Maas-Bauer, Claudia Wehr

**Affiliations:** 1 Department of Medicine I/Hematology, Oncology and Stem Cell Transplantation, Medical Center - University of Freiburg, Faculty of Medicine, University of Freiburg, Freiburg, Germany; 2 Institute of Medical Biometry and Statistics, University of Freiburg, Freiburg, Germany

**Keywords:** Cancer, Cancer stem cells

## To the Editor:

Allogeneic hematopoietic cell transplantation (allo-HCT), a potentially curative treatment for high-risk haematological malignancies and severe blood disorders, is often complicated by acute graft-versus-host disease (aGvHD), a severe, life-threatening condition primarily affecting the skin, gastrointestinal tract, and liver [1]. Immune responses, including those critical in aGvHD, are influenced by endogenous circadian rhythms [2]. Circulating T cell numbers, for instance, exhibit robust circadian variation, and clock genes modulate lymphocyte activation and cytokine production [3, 4]. Recent work by Hou et al. has demonstrated a significant association between earlier daytime stem cell infusion and reduced aGvHD risk, suggesting that the timing of stem cell infusion could potentially influence subsequent aGvHD development [5]. We aimed to validate these findings in a distinct, large, homogenous cohort of adult patients undergoing matched unrelated donor (MUD) allo-HCT, receiving a uniform, ATLG-containing GvHD prophylaxis regimen.

This single-centre, retrospective cohort study was conducted at the University Medical Center Freiburg, Germany. We included adult patients undergoing their first allo-HCT between March 2003 and August 2024. To minimize confounding, we only included patients receiving a G-CSF-mobilized peripheral blood stem cell graft from a 10/10 MUD with the following GvHD prophylaxis regimen: rabbit anti-T-Lymphocyte globulin (ATLG, ATG Grafalon®, formerly Fresenius®, 30 mg/kg), cyclosporine A and mycophenolate. Data were retrieved from a prospectively maintained internal transplant database.

The primary exposure was the time of day of allo-HCT defined by the arrival time of the stem cell product at the transplant ward immediately prior to transplantation. Patients were dichotomized into two groups: “early” infusion (arrival before 2:05 p.m.) and “late” infusion (arrival at or after 2:05 p.m.) according to median time of arrival.

The primary outcome was the cumulative incidence of aGvHD within 100 days post-transplant. aGvHD was diagnosed according to published guidelines [6]. Secondary outcomes included the time to neutrophil engraftment (first of three consecutive days with absolute neutrophil count ≥0.5 × 10⁹/L) and platelet engraftment (first of seven consecutive days with platelet count ≥20 × 10⁹/L without transfusion support).

The cumulative incidence function (CIF) was used to estimate the probability of aGvHD, accounting for death as a competing risk. Differences between CIF curves were assessed using Gray’s test. Fine and Gray proportional subdistribution hazards model [7] was employed, adjusting for potential confounders (infusion timing, patient age, donor age, diagnosis, remission status, gender match, TCI-Score, year of transplantation, infused CD34+ and CD3+ cell count/kg bodyweight). Statistical significance was defined as a two-sided *p*-value < 0.05. Analyses were performed using STATA/BE version 18.0 (StataCorp LLC, College Station, TX, USA).

**Fig. 1:**
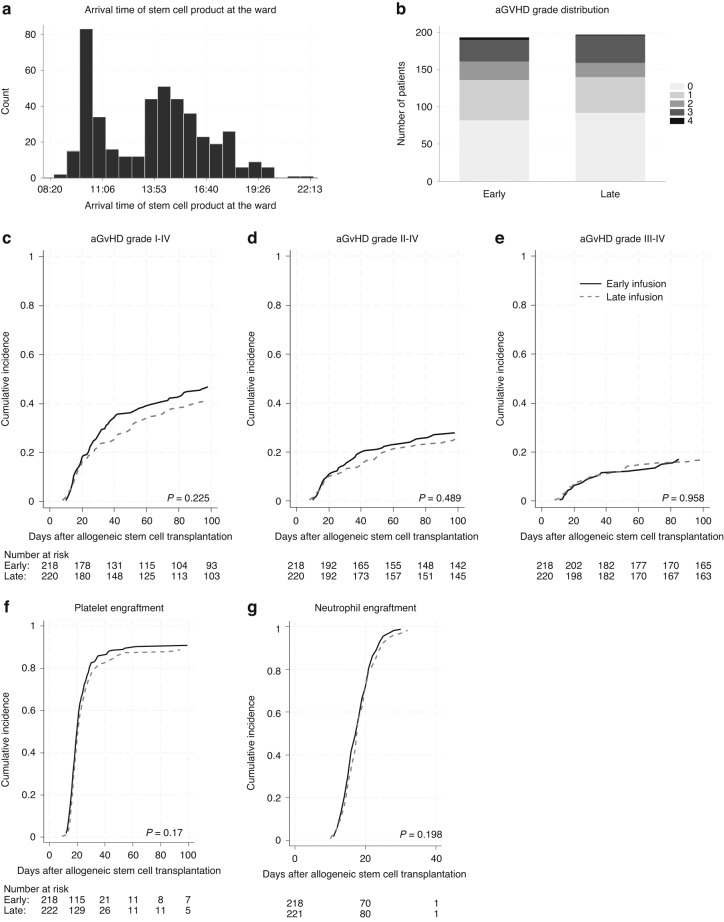
Effect of infusion timing on transplant outcomes. **a** Histogram showing the frequency distribution of stem cell product arrival times at the transplant ward (*N* = 440). **b** Stacked bar chart showing the distribution of maximum acute GvHD grades (0–4) by infusion timing group. **c** Cumulative incidence curves comparing early (<02:05 p.m. solid line) versus late (≥02:05 p.m., dashed line) stem cell infusion timing, accounting for death as competing risk. Panels show incidence up to day 100 for Grade I-IV aGvHD, **d** Grade II-IV aGvHD **e** Grade III-IV aGvHD, **f** Platelet Engraftment, **g** Neutrophil Engraftment. *P*-values calculated using Gray’s test.

Of the 440 included patients, 218 patients (49.5%) were allocated to the ‘early’ and 222 (50.5%) to the ‘late’ infusion group. The distribution of stem cell product arrival times at the ward (Fig. 1a) showed a bimodal pattern, with frequency peaks in the late morning (around 10:00–11:00 a.m.) and mid-afternoon (around 01:30 p.m.–04:40 p.m.).

Baseline patient, donor, and transplant characteristics were balanced (Supplementary Table 1). However, significant differences were observed between the early and late infusion groups for median donor age (27 vs 28 years, *p* = 0.014) and median year of transplantation (2020 vs 2016, *p* < 0.001). These variables were included as covariates in subsequent multivariate analyses.

The distribution of maximum aGvHD grades within 100 days was similar between early and late infusion groups (Fig. 1b). Cumulative incidence curves were generated to compare the incidence of aGvHD in patients receiving early versus late stem cell infusions (Fig. 1c–e). The cumulative incidence of aGvHD at day 100 post-transplant did not significantly differ between the early and late infusion groups. The incidence of grade I-IV aGvHD at day 100 was 47.2% (95% CI: 40.5–53.7%) in the early versus 41.8% (95% CI: 35.3–48.2%; *p* = 0.225) in the late group, II-IV aGvHD was 28.4% (95% CI: 22.0–35.1%) versus 25.5% (95% CI: 19.6–31.8%; *p* = 0.489), and for grade III-IV aGvHD it was 17.5% (95% CI: 12.5–23.2%) versus 16.8% (95% CI: 11.9–22.6%; *p* = 0.958).

Fine and Gray competing risk regression modelling was employed to quantify the effect of infusion timing on aGvHD incidence. Competing risk regression revealed no significant association between infusion timing and the subdistribution hazard for developing aGvHD (Supplementary Table 2). The adjusted hazard ratios for late versus early infusion were 0.93 (95% CI: 0.57–1.52; *p* = 0.78) for grade II-IV and 1.31 (95% CI: 0.72–2.38; *p* = 0.37) for grade III-IV aGvHD. Among the covariates, only a higher infused CD34+ cell dose showed a statistically significant association, conferring a reduced risk across all grades of aGvHD (Supplementary Table 2).

To ensure our primary findings were not solely dependent on the 02:05 p.m. cut-off, sensitivity analyses were performed. Using alternative cut-offs (01:00 p.m., 03:00 p.m.) also yielded non-significant results (Supplementary Table 3).

Infusion timing did not significantly impact hematopoietic engraftment kinetics (Fig. 1f, g). The cumulative incidence of neutrophil engraftment at day 30 was 98.6% (95% CI: 95.8–99.5%) in the early group versus 98.2% (95% CI: 95.2–99.3%) in the late group. The cumulative incidence of platelet engraftment at day 100 was 90.8% (95% CI: 86.1%–93,9%) in the early group and 88.7% (95% CI: 83.8–92.2%) in the late group.

Recent work by Hou et al. suggested earlier daytime stem cell infusion impacts aGvHD incidence and severity in related/haploidentical donor cohorts [5]. Our analysis of 440 MUD allo-HCT recipients found no statistically significant association between stem cell infusion timing and aGvHD cumulative incidence or severity. This divergence likely stems from critical population differences: our study population was restricted to MUD grafts with standardized ATLG/CSA/MPA prophylaxis, while Hou et al.‘s cohorts used related/haploidentical donors with heterogeneous prophylaxis. These donor types carry different baseline risks and immunological dynamics for aGvHD [8, 9].

Our null findings contribute to a critical perspective on the hypothesis that the specific daytime of administration significantly drives complex clinical outcomes like aGvHD. While circadian regulation of immunity is biologically established [10], extrapolating this to a clinical effect solely based on infusion timing warrants caution. Allo-HCT is a complex intervention influenced by numerous factors with infusion timing being just one variable. Methodological limitations of retrospective data are amplified by arbitrary time cutoffs. Unmeasured confounders correlated with time of day (e.g., patient acuity, logistics) are substantial and rarely fully accounted for [11].

Our study has limitations that must be acknowledged. Its retrospective, single-centre design limits generalizability. We used the arrival time of the graft on the ward as a proxy for infusion start time, which may introduce minor inaccuracies. Despite careful adjustment, residual confounding due to unmeasured variables (e.g. timing of stem cell apheresis, transport of the graft) cannot be excluded. However, with the knowledge that CD34+ cell count was a protective factor against GvHD and the fact that vital CD34+ cell counts decline with prolonged transport of graft [12], we advise against delaying allo-HCT based on the current evidence available on circadian influences.

Within our MUD allo-HCT cohort receiving ATLG-based GvHD prophylaxis, we found no evidence that stem cell infusion timing significantly influences aGvHD risk or engraftment. Our findings suggest logistical factors can likely guide infusion scheduling without compromising aGvHD risk, at least in our setting. Future research should prioritize prospective, randomized multi-centre studies encompassing diverse donor types to assess circadian immune modulation on transplant outcomes.

## Supplementary information

Supplemental Material

## Data Availability

The data that support the findings of this study are available from the corresponding author upon reasonable request.

## References

[1] Zeiser R, Blazar BR. Acute graft-versus-host disease - biologic process, prevention, and therapy. N Engl J Med. 2017;377:2167–79. 10.1056/NEJMra1609337. 29171820 10.1056/NEJMra1609337 PMC6034180

[2] Wang C, Lutes LK, Barnoud C, Scheiermann C. The circadian immune system. Sci Immunol. 2022;7:eabm2465. 10.1126/sciimmunol.abm2465. 35658012 10.1126/sciimmunol.abm2465

[3] Cermakian N, Labrecque N. Regulation of cytotoxic CD8+ T cells by the circadian clock. J Immunol. 2023;210:12–8. 10.4049/jimmunol.2200516. 36542828 10.4049/jimmunol.2200516

[4] Wang C, Zeng Q, Gül ZM, Wang S, Pick R, Cheng P, et al. Circadian tumor infiltration and function of CD8+ T cells dictate immunotherapy efficacy. Cell. 2024;187:2690–2702.e17. 10.1016/j.cell.2024.04.015. 38723627 10.1016/j.cell.2024.04.015

[5] Hou Y, Wu Y, Cao Y, Hu X, Sun Y, Wang H, et al. Optimizing stem cell infusion timing in the prevention of acute graft-versus-host disease. Cell. 2025;188:3030–3044.e17. 10.1016/j.cell.2025.03.022. 40168995 10.1016/j.cell.2025.03.022

[6] Harris AC, Young R, Devine S, Hogan WJ, Ayuk F, Bunworasate U, et al. International, multicenter standardization of acute graft-versus-host disease clinical data collection: a report from the Mount Sinai Acute GVHD International Consortium. Biol Blood Marrow Transpl. 2016;22:4–10. 10.1016/j.bbmt.2015.09.001. 10.1016/j.bbmt.2015.09.001 PMC4706482 26386318

[7] Fine JP, Gray RJ. A proportional hazards model for the subdistribution of a competing risk. J Am Stat Assoc. 1999;94:496–509. 10.1080/01621459.1999.10474144.

[8] Jagasia M, Arora M, Flowers MED, Chao NJ, McCarthy PL, Cutler CS, et al. Risk factors for acute GVHD and survival after hematopoietic cell transplantation. Blood. 2012;119:296–307. 10.1182/blood-2011-06-364265. 22010102 10.1182/blood-2011-06-364265 PMC3251233

[9] Meyer T, Maas-Bauer K, Wäsch R, Duyster J, Zeiser R, Finke J, et al. Immunological reconstitution and infections after alloHCT - a comparison between post-transplantation cyclophosphamide, ATLG and non-ATLG based GvHD prophylaxis. Bone Marrow Transpl. 2025;60:286–96. 10.1038/s41409-024-02474-1. 10.1038/s41409-024-02474-1 PMC11893447 39562716

[10] Zeng Y, Guo Z, Wu M, Chen F, Chen L. Circadian rhythm regulates the function of immune cells and participates in the development of tumors. Cell Death Discov. 2024;10:199. 10.1038/s41420-024-01960-1. 38678017 10.1038/s41420-024-01960-1 PMC11055927

[11] Özdemir BC, Bill R, Okyar A, Scheiermann C, Hayoz S, Olivier T. Chrono-immunotherapy as a low-hanging fruit for cancer treatment? A call for pragmatic randomized clinical trials. J Immunother Cancer. 2025;13; 10.1136/jitc-2024-010644 10.1136/jitc-2024-010644 PMC11877229 40032603

[12] Antonenas V, Garvin F, Webb M, Sartor M, Bradstock KF, Gottlieb D. Fresh PBSC harvests, but not BM, show temperature-related loss of CD34 viability during storage and transport. Cytotherapy. 2006;8:158–65. 10.1080/14653240600620994. 16698689 10.1080/14653240600620994

