# Supplementary material for: Influence of daytime of allogeneic stem cell transplantation on incidence of acute graft vs. host disease: a retrospective analytical cohort study
Source: Bone Marrow Transplant. 2025 Aug 2;60(10):1413–5. doi: 10.1038/s41409-025-02689-w (PMC12568623; doi:10.1038/s41409-025-02689-w)
Supplement: Supplementary file 1 — Supplemental Material [file 41409_2025_2689_MOESM1_ESM.pdf]

		early infusion N=218	late infusion N=222	p-value
**Patient age**	(median and range)	61(19-78)	50 (19-76)	0.15
**Donor age**	(median and range)	27 (18-50)	28 (18-56)	0.014
**Patient gender**	female	117 (53.7%)	122 (55.0%)	0.85
	male	101 (46.3%)	100 (45.0%)	
**Sex match**	match	124 (56.9%)	126 (56.8%)	0.97
	male donor to female recipient	69 (31.7%)	69 (31.1%)	
	female donor to male recipient	25 (11.5%)	27 (12.2%)	
**Year of transplant**	(median and range)	2020 (2005-2024)	2016 (2003-2024)	<0.001
**Blood group matching**	match	115 (52.8%)	105 (47.3%)	0.24
	major missmatch	49 (22.5%)	52 (23.4%)	
	minor missmatch	38 (17.4%)	54 (24.3%)	
	bidirectional missmatch	16 (7.3%)	11 (5.0%)	
**CMV-Status**	P+/D-	13 (6.0%)	16 (7.2%)	0.13
	P-/D+	44 (20.2%)	51 (23.0%)	
	P+/D+	84 (38.5%)	62 (27.9%)	
	P-/D-	77 (35.3%)	93 (41.9%)	
**Diagnosis**	MM	12 (5.6%)	16 (7.4%)	1.00
	AML	108 (50.2%)	105 (48.6%)	
	ALL	17 (7.9%)	17 (7.9%)	
	MDS	26 (12.1%)	24 (11.1%)	
	T-NHL	9 (4.2%)	12 (5.6%)	
	B-NHL	16 (7.4%)	14 (6.5%)	
	HL	1 (0.5%)	1 (0.5%)	
	MPN	16 (7.4%)	17 (7.9%)	
	SAA	5 (2.3%)	4 (1.9%)	
	Miscellaneous	5 (2.3%)	6 (2.8%)	
**Remission status**	steady disease	131 (60.1%)	110 (50.5%)	0.054
	active disease	87 (39.9%)	108 (49.5%)	
**TCI-Score**	(median and range)	2.5 (1.5-4)	2.5 (1.5-4)	0.12
**HCT-CI Score**	(median and range)	3 (0-10)	3 (0-10)	0.33

Supplementary Table 1 Baseline Patient, Donor, and Transplant Characteristics Stratified by Infusion Timing (Early vs. Late). Continuous variables were reported as median and range and compared using the Wilcoxon rank-sum test. Categorical variables were reported as frequencies and proportions (%), compared using the Chi-square (χ^2) or Fisher's exact test. R=Recipient, D=Donor, MM= Multiple Myeloma, AML= Acute myeloid leukaemia, ALL=Acute lymphoblastic leukaemia, MDS= Myelodysplastic syndrome, T-NHL=T-Non-Hodgkin-Lymphoma, B-NHL= B-Non-Hodgkin-Lymphoma, HL= Hodgkin's lymphoma, MPN= Myeloproliferative neoplasm, SAA= Severe anaplastic anaemia.

	Univariate Analysis		Multivariate Analysis	
**aGvHD I-IV**	SHR (95% CI)	p-value	SHR (95% CI)	p-value
Late daytime of Transplantation	0.84 (0.64-1.1)	0.225	0.97 (0.68-1.38)	0.873
CD34+ cell count /kg BW infused	0.95 (0.89-1.01)	0.078	0.94 (0.89-1.01)	0.034
**aGvHD II-IV**				
Late daytime of Transplantation	0.87 (0.6-1.3)	0.489	0.93 (0.57-1.52)	0.78
CD34+ cell count /kg BW infused	0.9 (0.81-1.0)	0.047	0.90 (0.82-0.99)	0.027
**aGvHD III-IV**				
Late daytime of Transplantation	1.01 (0.62-1.66)	0.958	1.31 (0.72-2.38)	0.374
CD34+ cell count /kg BW infused	0.87 (0.75-1.01)	0.069	0.84 (0.73-0.96)	0.01

Supplementary Table 2 Univariate and multivariate competing risk analysis of aGvHD I-IV accounting for infusion timing, patient age, donor age, gender match, diagnosis, remission status, infused CD34+ cell count per kg BW, infused CD3+ cell count per kg BW, TCI-Score and year of transplantation. Statistically significant covariates (CD34+ per kg BW) are displayed. Subdistribution hazard ratio (SHR). Confidence interval (CI).

	Grade I-IV		Grade II-V		Grade III-IV	
	SHR (95% CI)	p-value	SHR (95% CI)	p-value	SHR (95% CI)	p-value
**1 p.m. cut off**	1.04 (0.74-1.47)	0.8	0.88 (0.55-1.4)	0.59	1.23 (0.69-2.2)	0.47
**3 p.m. cut off**	0.81 (0.55-1.2)	0.27	0.96 (0.56-1.63)	0.85	1.28 (0.64-2.57)	0.48

Supplementary Table 3 Sensitivity Analysis: Competing risk regression for aGvHD incidence using alternative time cutoffs (01:00 p.m. and 03:00 p.m.) for late vs. early transplantation. Subdistribution hazard ratio (SHR). Confidence interval (CI).
